# MXene/TiO_2_ Photocatalyst: The Key Role of MXene Electron Trapping in Water and Air Treatment

**DOI:** 10.3390/ijms27093975

**Published:** 2026-04-29

**Authors:** Áron Ágoston, Laura Lakatos, Ágota Deák, Gergő Ballai, Karolina Solymos, Szabolcs Kocsis Szürke, László Janovák, Ákos Kukovecz, Zoltán Kónya, Zsolt Pap

**Affiliations:** 1 Department of Physical Chemistry and Materials Sciences, University of Szeged, Aradi v.sqr.1, H-6720 Szeged, Hungary; janovakl@chem.u-szeged.hu; 2 Interdisciplinary Excellence Center, Department of Physical Chemistry and Materials Science, University of Szeged, Rerrich Béla sqr. 1, H-6720 Szeged, Hungary; 3 Department of Applied and Environmental Chemistry, University of Szeged, Rerrich Béla sqr. 1, H-6720 Szeged, Hungary; laura.lakatos@chem.u-szeged.hu (L.L.); ballaig@chem.u-szeged.hu (G.B.); kakos@chem.u-szeged.hu (Á.K.); konya@chem.u-szeged.hu (Z.K.); pzsolt@chem.u-szeged.hu (Z.P.); 4 Department of Physical and Environmental Geography, University of Szeged, Egyetem Str. 2-6, H-6722 Szeged, Hungary; solymoskarolina@geo.u-szeged.hu; 5 Central Campus Győr, Széchenyi István University, Egyetem sqr. 1. 303., H-9026 Győr, Hungary; kocsis.szabolcs@ga.sze.hu; 6 Center 3B, Laboratory of Advance Hydrobiology and Biomonitoring, Babeş-Bolyai University, Clinicilor 5-7, R-400015 Cluj-Napoca, Romania; 7 Hungarian Department of Biology and Ecology, Faculty of Biology and Geology, Babeş-Bolyai University, Republicii 44, R-400015 Cluj-Napoca, Romania

**Keywords:** MXene, titanium dioxide, composite photocatalyst, air purification, water treatment, electron trapping, ALD

## Abstract

The photocatalytic activity of TiO_2_ can be increased by incorporating it into a composite with an electron-trapping co-catalyst. MXene can perform this task as an electron-conducting material. In addition to trapping electrons, it also affects the defects in TiO_2_ near the interface. To screen for the best photocatalytic performance, three types of composites were prepared: by physical mixing, chemical deposition, and ALD. During characterization, the structural, optical, and photoelectrochemical properties were determined. Photocatalytic activity was examined in suspension (phenol conversion) and on a layer (gas phase ethanol conversion). It was found that the composite containing the lowest proportion of cocatalyst (1 wt.%) had the highest photocatalytic activity. According to the results of photocatalytic activity measured in suspension, the physical mixtures were proven to be more effective than neat TiO_2_, with the composites converting approximately the total amount of phenol in ~40 min, while TiO_2_ required ~80–90 min to do so under the same conditions. Thus, the electron-trapping role of MXene is clearly demonstrated in suspension applications, which is also confirmed by other characterization methods (photoluminescence, photocurrent density). TiO_2_ performed best during ethanol conversion, as it has the highest ethanol adsorption capacity (33.41%). During ethanol conversion tests, the MXene electron-trapping property was most effectively demonstrated in composites formed using the ALD method.

## 1. Introduction

In photocatalysis the most frequently investigated material is TiO_2_, due to its inertness, stability, low cost and availability in consistent quality/quantity [1,2]. Many researchers are working on developing this material and are constantly investigating its potential uses. Despite its impressive properties, there are also some drawbacks that should be addressed in the case of this material (wide band gap, rapid charge recombination) [3,4,5,6]. There are several approaches that can be used to enhance the photocatalytic activity of a material under certain conditions. One suitable technique is composite formation, in which another material, known as a cocatalyst, is added [7]. This material does not necessarily possess photocatalytic activity, but it can affect the main catalyst in different ways. For example, it traps excited electrons, thereby suppressing recombination and allowing the positive holes more time and opportunity to be utilized (by oxidizing the donor molecule).

Cocatalysts can be conductors (MXenes, graphene, etc.) or metal oxides that can accept electrons (WO_3_, V_2_O_5_) [8,9,10]. Another method for increasing the photocatalytic efficiency is noble metal deposition (Ag, Pt, Rh) on the surface, which can promote the adsorption of pollutants and influence optical properties (local surface plasmon resonance or local changes in charge density) [11,12]. Another technique used to modify photocatalysts is doping, which involves introducing foreign elements into the lattice of the photocatalyst. Doping affects the structure of the crystal lattice, the optical properties of the material, while introducing new discrete energy levels into the band structure. Almost any element can be used as a doping element: transition metals (Fe, Ni, Ag), metals (Al, Ga), and non-metals (N, S, C) were successfully used [13,14,15]. A less commonly used technique for modifying photocatalysts is changing their morphology [16], e.g., specific surface are, porosity, crystal facet engineering [17,18].

MXenes are currently quite popular materials and are investigated intensively [19,20,21,22,23,24]. In terms of composition, they are metal carbides, nitrides, or borides; of the three groups mentioned, carbide is the most common. Usually MXenes contain one metal (M_2_C, M_3_C_2_, and M_4_C_3_), which is usually titanium, but there are also some examples containing molybdenum (Mo_2_C, Mo_3_C_2_) or chromium (Cr_2_CT_x_), where T is a surface group (oxide, hydroxyle, fluoride) [25]. In addition, there are also bimetallic MXenes, which vary in stoichiometry and composition and may contain a number of transition metals (Ti, Co, Mo, Cr, V). They are layered 2D materials, consisting of multiple sheets [25]. The stability shown by MXenes is acceptable. It is known that nanoparticles can be inserted between the sheets (e.g., Fe_3_O_4_, [26]).

This family of materials is particularly promising for use in electronics and sensors (due to their good electrical conductivity). In vivo and in vitro applications are also known, as they are considered non-toxic [27,28]. A growing number of studies are investigating the applicability of MXenes in water purification, particularly when incorporated in membranes [29,30]. It is also a suitable cocatalyst for trapping photoelectrons originating from the excited photocatalyst [31]. MXene has been successfully used in photocatalyst composites, for example in the conversion of dye pollutants (decolorization) or CO_2_ reduction [32,33].

Nowadays, there is a growing emphasis on developing photocatalysts suitable for industrial applications, with the aim of using them for environmental remediation or the degradation of industrial byproducts [34,35,36]. Beyond scaling up photocatalyst production, numerous industrial factors must be considered to ensure cost-effectiveness. Using photocatalysts in coatings to neutralize pollutants (air purification) is significantly more economical than employing them in suspension-based systems [37,38]. For photocatalytic surfaces, the choice of binder, the coating formation process, and long-term coating stability are critical parameters. A TiO_2_–MXene composite may exhibit the necessary properties for effective performance under real-world conditions. Due to the hydrophilic nature of TiO_2_, it forms a stable aqueous suspension, enabling straightforward coating deposition—for example, via simple spray-coating. Even with a certain MXene content, the composite generally retains its hydrophilicity, especially when TiO_2_ particles fully surround the MXene sheets. With an appropriate water-based binder and by optimizing suspension parameters (such as pH and ionic strength), a long-term stable coating material can be produced in which the active photocatalyst component is a TiO_2_–MXene composite. This study does not directly examine scalability or usability in real-world environments; however, it provides the foundation for a larger project whose final goal is to clean the environment described in this paragraph. The first step is to develop a suitable photocatalyst, whose mechanism and effects are already known. This article focuses on that step.

In this work, MXene and titania-based composites were investigated. The effect of the composite preparation methodology and the MXene content was investigated on the photocatalytic activity of TiO_2_. The composite synthesis strategies were: physical mixing (TiO_2_+MXene); sol–gel synthesized TiO_2_ (synthesis in the presence of MXene); and TiO_2_ deposited MXene (using ALD—atomic layer deposition). The photocatalytic activity of the prepared composites were analysed in different scenarios: liquid/solid interface analysis (phenol conversion) and gas/solid interface analysis for modelling VOC pollution (ethanol conversion).

## 2. Results

### 2.1. Characterisation

**Figure 1:**
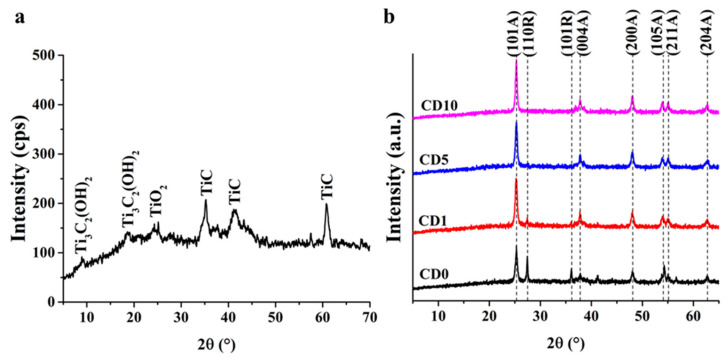
XRD patterns of the synthesized MXene (**a**) and the samples synthesized by chemical deposition (**b**).

Figure 1a shows the diffractogram of the prepared MXene. The diffraction peaks at 35.24°, 41.2°, and 60.84° belong to TiC, the diffraction peak at 25.12° belongs to TiO_2_, and the diffraction peaks at 18.56° and 9.84° belong to Ti_2_C_3_ MXene containing terminal hydroxide groups (Ti_3_C_2_(OH)_2_). JCPDS card no. 73-0472 was used for the diffraction identification of Ti_3_C_2_, JCPDS card no. 52-0875 was used for the diffraction identification of Ti_3_C_2_(OH)_2_, and JCPDS card no. 32-1383 was used for the identification of TiC.

Figure 1b shows the diffractogram of MXene/TiO_2_ composites produced by chemical deposition (PMX samples). All detectable diffraction belongs to TiO_2_. The (101), (004), (200), (105), (211) and (204) diffractions belong to the anatase phase; the (110) and (101R) diffractions belong to the rutile phase. JCPDS card no. 21-1272 and JCPDS card no. 21-1276 were used for the diffraction identification of TiO_2_. By increasing the proportion of MXene, the rutile phase disappears, and anatase crystallization becomes preferred; due to the surface energy conditions of MXene, the lattice parameters may change during calcination.

**Figure 2:**
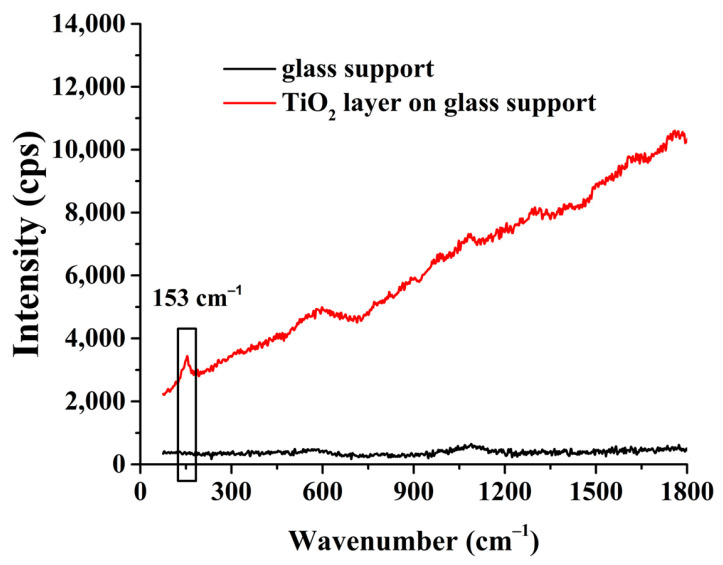
The Raman spectrum of the glass support and the TiO_2_ layer on glass support.

The successful layer formation using the TiO_2_ ALD method and the presence of the layer were confirmed by Raman measurements. Spectra were recorded in the fingerprint range (Figure 2). It can be seen that a peak appeared at a wavenumber of 153 cm^−1^ on the glass substrate carrying the TiO_2_ layer; this peak is the Raman shift of the symmetric Ti-O stretching vibration bond (black box on the figure). In the literature, a value of ~144 cm^−1^ is usually measured for pure anatase TiO_2_. In our case, the slight blue shift is due to surface conditions. A very thin TiO_2_ layer was measured, which was bonded to a carrier material, so the surface structure may change, and stress may occur. Another frequently observed cause is phonon confinement, which occurs when the crystallite size is too small (<~10 nm) [39]. The mass of the separated TiO_2_ layer is ~50 mg, so the coverage is the same as that of MXene, 0.5 mg·cm^−1^, and the content of composite TiO_2_ and MXene is also 50%.

During EDX measurements, as well as during Raman measurements, the glass substrate was measured without TiO_2_ and with a TiO_2_ layer deposited on the surface using the ALD method. The pure glass substrate measured 0% Ti, and after ALD treatment (3000 steps/layer) it measured 59.50%. Measurements were performed at magnifications of 50× and 250×. This proves the successful separation of TiO_2_. The exact same percentage of Ti was determined at both magnifications, proving that perfectly homogeneous TiO_2_ can be separated using this method even with a relatively large number of ALD cycles. The image of the measured surface and the ED spectrum of the elemental composition determined during the measurement can be seen in Figure S1 in the Supplementary Material.

**Figure 3:**
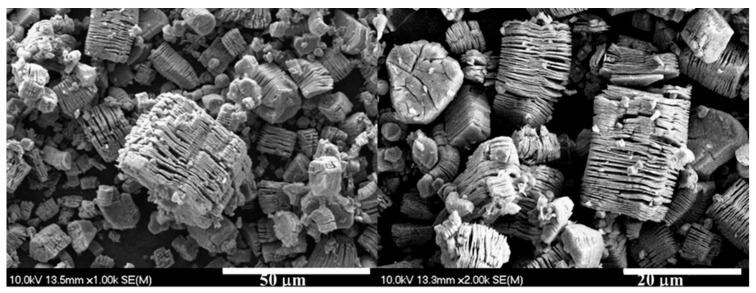
SEM images of the produced MXene.

SEM images were taken to verify the layered structure of the MXene produced. Figure 3 shows proof of the successful synthesis.

**Figure 4:**
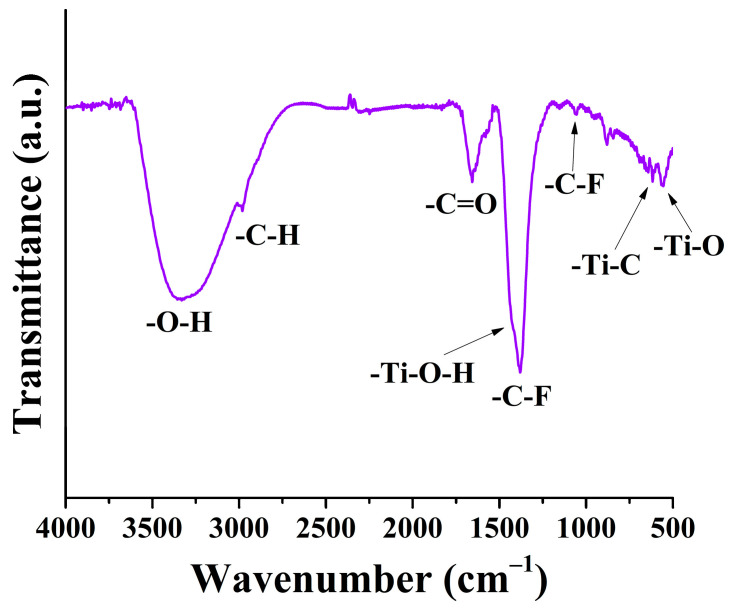
FTIR spectrum of the synthesized MXene.

FT-IR measurements identified vibrations associated with various surface groups (Figure 4), which can be used to characterize the surface of the synthesized MXene. This is important because surface groups influence numerous material properties and interfacial phenomena. The following vibrations were identified: the signal between 3650 and 2630 cm^−1^ corresponds to the –OH vibration [40]; the signal at 2980 cm^−1^ is the –C-H bond [41]; the signal between 1780 and 1525 cm^−1^ corresponds to the –C=O vibration [42,43]; the signal between 1500 and 1200 cm^−1^ corresponds to the –C-F vibration [44]; the signal at 1430 cm^−1^ is the –Ti-O-H group [43]; the signal at 1060 cm^−1^ is the –C-O group [43]; the signal at 617 cm^−1^ is the –Ti-C bond [43]; and the signal at 555 cm^−1^ is the –Ti-O bond [43].

The specific surface area of the produced MXene, commercial TiO_2_ and CD samples was determined by N_2_ adsorption, and the BET method was used for evaluation; the results are in Table 1, and the linearized graphs used for the calculation are shown in Figure S2. In the case of CD samples, the specific surface area decreases primarily for mathematical reasons. As the MXene content increases, the density of the composite also rises, while the specific surface area is normalized to mass. Consequently, when a higher-density component is added to the composite, its contribution to the total mass becomes proportionally larger than its contribution to the surface area. This causes the denominator in the calculation to increase more significantly, leading to a lower specific surface area value. The specific surface areas need to be determined in order to interpret the results of the photocatalytic ethanol conversion tests.

**Figure 5:**
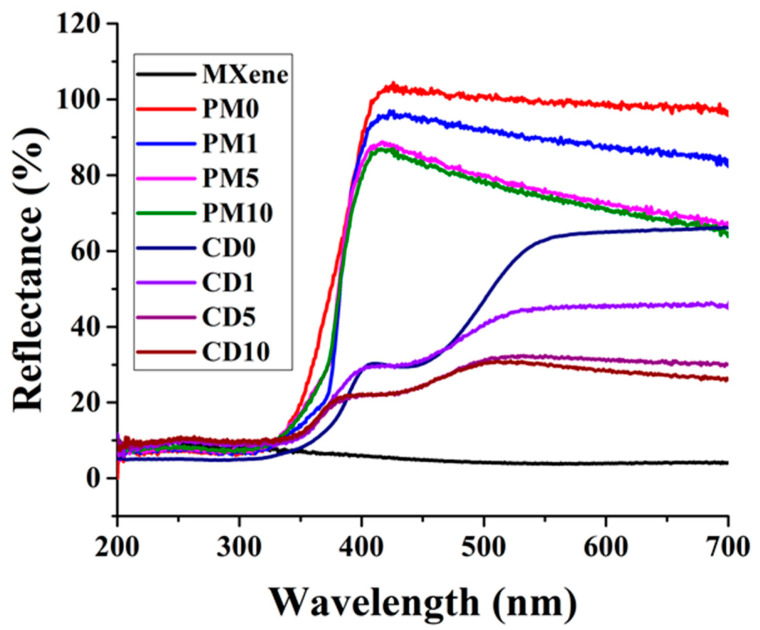
The DRS spectrum of the samples.

The diffuse reflectance spectrum of the samples is shown in Figure 5. The figures illustrating the determination of band gap are shown in the Supplementary (Figure S3). The shape of the spectra shows that the physical mixtures (PMX samples) match the TiO_2_ spectrum (PM0), with a slight increase in absorbance in visible light due to the addition of MXene, which is a black material that absorbs visible light. In terms of optical properties, the band gap of the composite sample prepared by physical mixing shifts slightly from 3.22 eV to 3.18 eV. This is due to band bending at the interface between TiO_2_ and MXene, which results from the different band structures of the two materials in direct contact (when two materials with different band structures come into contact, a local internal electric field is generated, causing the energy bands to bend at the interface). This occurs because the contact area between the two materials increases significantly during grinding, and many photons are reflected from the interface. In the case of samples prepared by chemical deposition (CDX), two structures can be identified. The band gap value of the TiO_2_ content of the samples suffered a blue shift (3.23–3.29) due to nitrogen doping, what can happen because of calcination in the N_2_ atmosphere [45]. Another band gap value was also observed, which also suffered a blue shift (2.20–2.47 eV). The detected band gap value explains the greenish colour of the samples. TiO_2_ has an anatase structure in all samples according to the band gap, as well as according to XRD.

**Figure 6:**
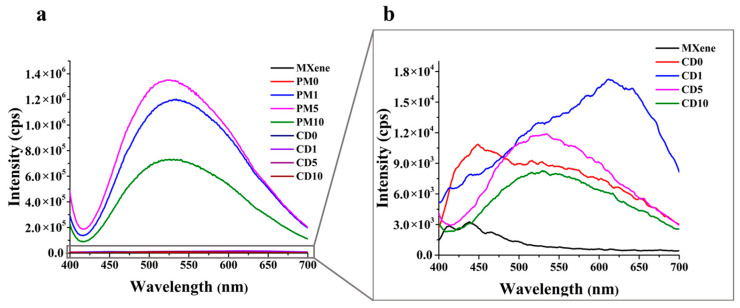
Photoluminescence spectrum of the TiO_2_, MXene, PMX and CDX samples (**a**) and magnified samples with lower photoluminescence intensity (**b**).

During PL measurements, the photon emission of the samples after excitation was examined; the results are shown in Figure 6. It is worth examining the samples within a series, as the excited electrons of different materials undergo different processes. Emissions measured at ~520 nm can be attributed to near-surface/bulk oxygen defects and self-trapping states in TiO_2_; this can also be observed in PM and CD samples [46]. In the case of PM samples, the emission intensity of samples containing MXene was higher than that of TiO_2_ (PM0). Since these materials form only physical mixtures, their behaviour is strongly governed by interfacial phenomena. The surface functional groups of MXene (as identified in the FTIR section: –F, –OH, –CH) interact with the TiO_2_ surface and its defect sites through electrostatic interactions, thereby influencing the utilization of TiO_2_ surface defects [47]. MXene can act as an electron trap (Schottky junction), the photoexcited electrons from TiO_2_ are able to transfer into the conduction band of MXene; this process is facilitated by the band bending observed in the DRS measurements [48]. In physical mixtures, however, the contact between the two components is significantly less ideal than in a grown MXene–TiO_2_ heterostructure. As a result, electron transfer from TiO_2_ to MXene is less efficient. Consequently, electrons can accumulate at the levels corresponding to 520 nm electronic transition, where they undergo radiative recombination, leading to an increase in the measured emission intensity. It can be stated that the emissions from the PM10 sample are lower than those from the PM5 sample. This is because, although MXene has an electron-trapping effect according to measurements, only TiO_2_ can be excited with sufficient quantum efficiency, so the lower the proportion of TiO_2_ in the sample, the fewer excitons are generated, which may ultimately result in photon emission. In the case of composites produced by chemical deposition (CDX samples), the photoluminescence emission decreased with the decrease in the TiO_2_ ratio, comparable to TiO_2_ produced under similar conditions (CD0). Although MXene’s electron-trapping role can also be demonstrated here by the fact that the emission detectable at ~440 nm (surface oxygen vacancy) in TiO_2_ shifts towards the emission caused by the near-surface/bulk oxygen vacancy and self-trapping state defect detectable at ~520 nm, the intensity is so low because there are fewer oxygen vacancies in the sample, and it is less crystalline than commercial TiO_2_. This shift suggests that MXene exerted the same effect at the interface in these samples as it did in the physical mixtures. In the case of CD1, an emission around ~620 nm can be observed, which can be attributed to Ti^3+^ defects, as these are chemically deposited samples rather than commercial TiO_2_ with a fixed structure, so it is less likely that the MXene ratio used will affect the quality and quantity of the defects formed. It is more likely that the MXene ratio affects the excited electron recombination path (or excitation), and that emission from the Ti^3+^ defect is preferred over the CD1 sample [49]. This behaviour is observed in this sample because the MXene content is sufficiently low that its influence on defect sites outweighs its electron-trapping capability. In the PM1 sample, this effect is less pronounced because, as a physical mixture, the physical contact between the MXene particles is greater, which enhances their electron-conducting properties. In contrast, in the CD1 sample, the TiO_2_ matrix surrounds the MXene particles, effectively isolating them from each other. At higher MXene concentrations, a significant contact area between MXene particles is already observed even in the CD samples. This outstanding property may also explain why samples containing 1 wt% MXene have the best photocatalytic activity within their own series. When MXene content is low, electron trapping and the effect on defect utilization (modification of recombination pathways) occur to a comparable extent, and the two processes have a synergistic effect on each other.

**Figure 7:**
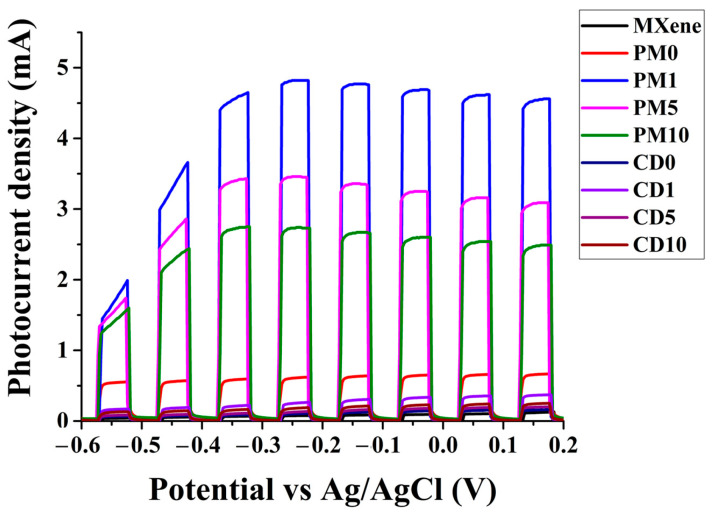
Photovoltammograms of MXene and composite samples.

Figure 7 shows the photovoltammograms recorded during the measurement of the photocurrent density for the different samples. The photocurrent density of physical mixture samples (PMX samples) increased significantly compared to that of TiO_2_ (PM0), so the electron-trapping role of MXene can be well measured using this method. The potential applied during the measurement enhances the electron-trapping function of the conductive component by facilitating the removal of electrons. The measured photo current density decreases continuously as the MXene content increases. This is because although MXene traps (removes) electrons, the TiO_2_ content of the samples decreases with increasing MXene content, and only TiO_2_ is excited to a significant extent in the composite. These results converge well with those observed during PL measurements and photocatalytic activity measurements. In the case of chemically deposited samples (CDX samples), much lower photocurrent densities were measured, but the same trend was observed (order within the series and ratio of photocurrent density values to each other) with the difference. All this suggests that physical composite formation is much more effective for electron trapping than the chemical separation composite formation we used.

### 2.2. Photocatalytic Activity

**Figure 8:**
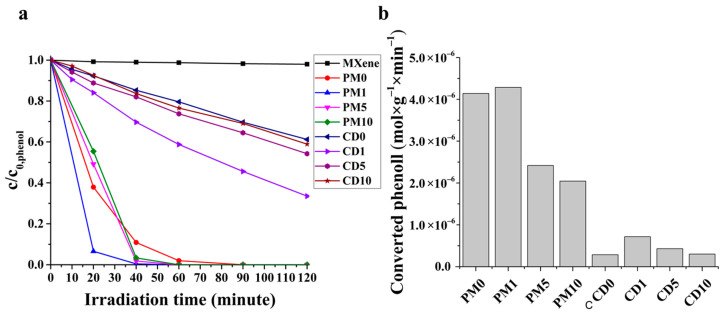
The conversion of phenol (**a**) and the specific converted phenol (**b**).

The photocatalytic activity determined through the photocatalytic conversion of phenol is shown in Figure 8. The photocatalytic activity of MXene used as a cocatalyst is negligible. In the case of physical mixture samples (PMX), increased photocatalytic activity can be measured compared to TiO_2_ (PM0), and the determined photocatalytic activity decreases with increasing MXene content in the composite. In 90 min, the total phenol content is converted in all four samples. In the case of samples containing MXene, phenol conversion reaches ~97% in 40 min, while in the case of TiO_2_ (PM0), it reaches only ~89%. Thus, a minimum MXene concentration shows the greatest effect for the reasons discussed earlier. Although it has electron-trapping properties, it remains only a cocatalyst, so its ratio in the composite cannot increase above a threshold, as there would then be too small a ratio of the photocatalytically active material (TiO_2_). Furthermore, at low MXene concentrations, the effect on the utilization of TiO_2_ defects is greatest, since electron transport is less efficient here than in samples containing a higher proportion of MXene. The order follows the order observed for photocurrent density. The order is different in PL measurements because the powder sample is measured in air; there is no measurable efficiency for the continuous acceptance of generated charge carriers, and recombination is the preferred process. During photocatalytic activity tests and photocurrent density measurements, the possibility of accepting the generated charge carriers is continuous. In the series of samples produced by chemical deposition (CDX), the MXene cocatalyst also increased photocatalytic activity compared to the produced TiO_2_ (CD0). The order and reasons are the same as those described for the physical mixture samples (PMX). In the case of CDX samples, none of the samples achieved 100% phenol conversion efficiency during the 120 min period investigated. CD1 had the best efficiency, ~67%, while the worst was the sample without MXene (CD0), ~39%. Figure 8b shows the specific amount of phenol converted in 40 min (normalized phenol conversion). The main consideration in selecting the specific time was to enable a clear comparison. Overall, it can be said that for suspension applications, the ideal MXene ratio in the composite is 1 wt.%, as this ratio provides enough MXene to utilize its electron-trapping role, but still enough of the photocatalytically active component (TiO_2_) for adequate efficiency, ensuring a large amount of excitable component and a relatively large amount of photocatalytically active surface sites.

Cycling tests were also carried out, with three cycles measured for each sample. The stability of the samples is compared based on the normalized phenol conversion results (Table 2). The decay curves are shown in Figure S4. In the case of PM samples, a slight but steady decrease in photocatalytic activity can be observed over repeated cycles. The behaviour of the CD samples is more complex: while CD0 exhibits a similar decreasing trend as the PM samples, the MXene-containing CD samples behave differently. During photocatalysis, the MXene surface can partially transform into anatase, effectively extending the existing TiO_2_ lattice [50]. This transformation enhances photocatalytic activity, as anatase is intrinsically highly active, and it simultaneously strengthens the chemical contact between the MXene and TiO_2_ phases by forming a heterostructure.

As a result of this phenomenon, the photocatalytic activity of CD1 remains nearly constant across the three cycles, whereas the activity of CD5 and CD10 increases with the number of cycles. At higher MXene contents, the MXene–TiO_2_ interfacial area is statistically larger, leading to the formation of more extensive heterostructures and thus improved photocatalytic performance.

**Figure 9:**
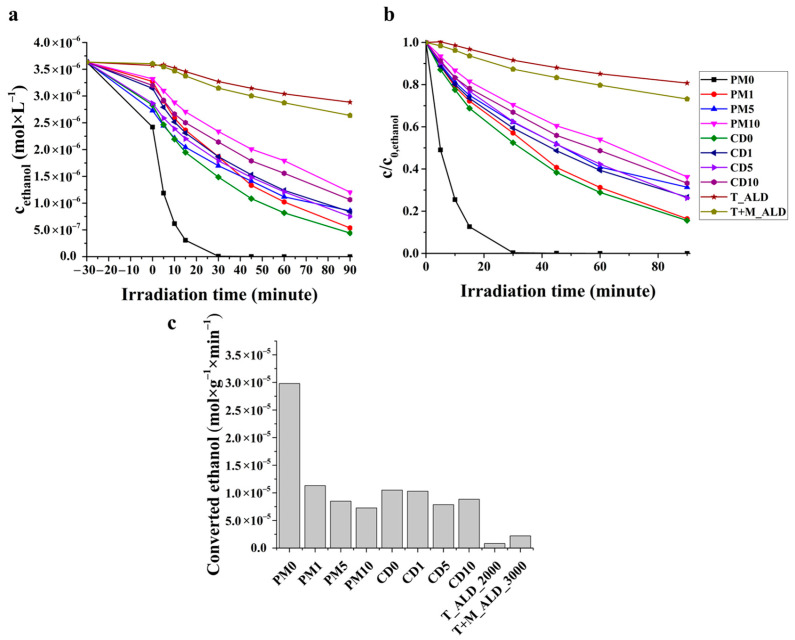
The conversion of ethanol with adsorption/desorption (**a**), relative change in concentration (**b**) and actually converted ethanol (**c**).

During photocatalytic ethanol conversion tests, it was found that none of the composites reached the efficiency level of TiO_2_ (PM0). By 30 min, all the ethanol had been completely removed from the steam space, but it can be said that within a series (PMX or CDX), increasing the cocatalyst ratio reduces photocatalytic activity (Figure 9). The ethanol conversion efficiency of the composite samples varied between ~20 and 100% over 90 min. In the case of PM samples, as the MXene ratio decreases, the photocatalytic ethanol conversion efficiency increases from ~64% to ~100%, and in the case of CD samples, as the MXene ratio decreases, the ethanol conversion efficiency also increases from ~66% to ~84%. The actual amount of ethanol converted is shown in Figure 9c. In both series, the sample containing 5 wt% MXene shows a local maximum due to its adsorption/absorption effect. A major difference compared to phenol conversion tests is that during ethanol conversion, photocatalytic activity is measured at the solid/gas interface, which limits the utilization of charge carriers, as there is no guarantee of being unlimited, and a continuous electron donor molecule (pollutant, medium) is not ensured on the hole side. In the case of suspension (solid/liquid interface), the medium, in our case water, is available without restriction. The surface of the photocatalyst is well hydrated (adsorption), mainly by hydroxide ions, which function as electron donors to the positively charged hole, generating hydroxyl radicals that are continuously consumed by the pollutant molecules. This process continues as long as there is water and pollutant molecules. During ethanol conversion experiments, water vapor is also present, forming a very thin layer on the surface of the photocatalyst, consisting of water and ethanol. During ethanol conversion, the amount of ethanol in this layer decreases and is refilled from the vapor phase. So, first, absorption occurs in the layer. This absorbed ethanol is then converted. So, in contrast to the suspension experiments, it is not the rate of hydroxyl radical production that is critical here but the absorption of the contaminant molecule, as the rate of absorption is lower. During the measurement of the 30 min adsorption period (absorption and adsorption), it was found that in the case of TiO_2_ (PM0), ~33% of the ethanol had already been absorbed onto the surface and surface layer before the photocatalytic test began. Therefore, among the samples tested, TiO_2_ (PM0) has the highest adsorption/absorption tendency, which explains why this sample is the most effective under the conditions applied. This is because pure TiO_2_ is the most hydrophilic, so the specific adsorbed water is the thickest, but since the specific surface area is also the largest in the case of TiO_2_ (50.3 m^2^·g^−1^), the layer on the surface of TiO_2_ is also the thickest in absolute terms. Due to the presence of MXene in the composites, the relative amount of TiO_2_ decreased, thus reducing the photocatalyst surface area (specific surface are of MXene is 4.6 m^2^·g^−1^). After 30 min of adsorption, we measured the ratio of ethanol remaining on the surface of the different samples, and the results are shown in Table 3. It can be seen that as the TiO_2_ ratio decreases, the amount of ethanol remaining in the vapor space increases, and the trend closely follows the ethanol conversion efficiency, except for samples PM5 and CD5, which show locally outstanding values. Due to the phenomenon mentioned above, MXene used as a cocatalyst can best exhibit its electron-trapping properties in suspension applications, where electron donor molecules are available in virtually unlimited quantities due to the medium. Cycling tests were also carried out, with three cycles measured for each sample. The stability of the samples is compared based on the normalized phenol conversion results (Table 2). The decay curves are shown in Figure S5. In general, it can be observed that the photocatalytic activity decreases more significantly between cycles 1 and 2 than between cycles 2 and 3; thus, the photocatalytic activity stabilizes after a small number of cycles.

In the case of composite samples prepared using the ALD method, it was also observed that the degree of adsorption/absorption decreased with the appearance of MXene. Despite its better adsorption/absorption properties, the sample containing MXene performed better in the photocatalytic ethanol conversion experiment. The ethanol conversion efficiency was ~19.3% for T-ALD and ~26.8% for T+M_ALD. In the case of composite samples prepared with ALD, the electron capture role of MXene is more demonstrable, probably due to the connection between the two components of the composite. In this case, TiO_2_ continuously crystallizes onto MXene, allowing sufficient time for each layer to crystallize, while in other composite formations, all TiO_2_ sources were added to MXene simultaneously in large quantities. Thus, in the case of ALD samples, the specific contact surface between crystalline TiO_2_ and MXene may be larger.

The two types of photocatalytic experiments are comparable in terms of the specific efficiency of the photocatalyst in terms of time and mass, the results of which are shown in Table 1.

## 3. Discussion

This chapter summarizes the observations and explanations presented earlier. Three types of TiO_2_–MXene composites were successfully synthesized using physical mixing (PM), chemical deposition (CD), and the ALD method. The goal of creating these composites is to achieve higher photocatalytic activity than that of TiO_2_ alone and to better understand the underlying mechanism. In the composites, TiO_2_ functions as the photocatalyst, while MXene serves solely as a cocatalyst that modifies the properties of TiO_2_. In the physical mixture, commercial p25 TiO_2_ was used, which contains both anatase and rutile phases; in the case of TiO_2_ produced by chemical deposition (sol–gel method), increasing the MXene content results in the formation of only the anatase phase beyond a certain threshold.

In a TiO_2_–MXene composite, the interface between the semiconducting TiO_2_ and the conductive MXene leads to the formation of a Schottky barrier, which induces band bending and facilitates the transfer of excited electrons from TiO_2_ into the conduction band of MXene. The presence of band bending was confirmed by DRS measurements, and a slight reduction in the band gap was also observed. In addition to acting as an electron trap, MXene influences the recombination pathway of the excited electrons in TiO_2_. Direct electron trapping is supported by the decrease in emission associated with direct recombination. The surface functional groups of MXene (–OH, –F, –CH) were identified using FTIR measurements, and these groups were found to interact with defects in TiO_2_ at the TiO_2_–MXene interface, primarily through electrostatic interactions. This effect was confirmed by PL measurements. In the case of PM samples, a significant increase was observed in the emission intensity associated with shallow defects (~520 nm, oxygen vacancies). This increase can be explained by the fact that, in PM samples, the contact area and the quality of the interfacial contact between TiO_2_ and MXene are not ideal. The interaction is not as strong as it would be if the two crystal phases were co-crystallized (intergrowing). As a result, electron transportation to MXene is not achieved efficiently. However, due to the electrostatic attraction exerted by interfacial groups, electrons are drawn toward the surface, where—if transfer to MXene cannot occur—they are highly likely to become trapped at shallow defect sites near the surface, from which radiative recombination becomes the preferred pathway. Thus, as confirmed by PL measurements, charge transfer is influenced by MXene in two ways: through electron trapping, which leads to complete charge separation, and through the modification of recombination pathways at defect sites. Both processes are understood to delay charge recombination, thereby statistically increasing the likelihood of charge-driven processes such as photocatalysis. In the case of the CD samples, a much larger contact surface area between TiO_2_ and MXene is assumed, since the MXene particles are surrounded by TiO_2_ and are calcined together with the sample. Evidence for this is also provided by the PL measurement results. For the CD1 sample, which contains the lowest MXene content, the main emission is detected around ~620 nm. This emission is attributed to Ti^3+^ defect sites on the TiO_2_ surface, and it appears with the highest intensity because the surface contact is greatest in this case. It is clearly shown that surface defect sites dominate, but a shoulder is also visible at ~520 nm, indicating that the previously mentioned emission is likewise detected and that the previously described phenomena also occur. In this sample series (CD), it is assumed that, due to calcination, a very thin layer is formed in which TiO_2_ and MXene are crystallized together into a heterostructure, through which electron transfer is facilitated. However, not all excited electrons are able to pass through this layer, so accumulation may occur within it. Therefore, emission from surface defects is observed to be the highest in the case of CD1. When the MXene ratio is further increased (CD5 and CD10), the emission from defects is no longer dominated by the ~620 nm level; instead, as with the PM samples, it is dominated by the ~520 nm level (shallow, near-surface defects). This is because, due to the higher MXene concentration, the contact between MXene particles is greater (and the TiO_2_–MXene interface area decreases specific), thereby enhancing electron transfer; consequently, direct recombination decreases, and excited electrons accumulate less at the surface compared to the CD1 sample. Among the detectable radiative recombinations, the one associated with shallow defects becomes prominent. The electron-trapping ability of MXene can also be directly demonstrated by the photocurrent density. The increase in the measured photocurrent can be explained by the fact that MXene has accepted the excited electron from TiO_2_, so the hole remaining on TiO_2_ oxidizes the water, thereby increasing the photocurrent. Due to the electric potential applied during the measurement, MXene is able not only to accept but also to conduct electrons, thereby demonstrating MXene’s conductivity as well.

The results of the photocatalytic experiments confirmed the findings from the material characterization measurements. In the phenol degradation tests, the composite samples showed better performance than TiO_2_ in both sample series, indicating that charge separation is enhanced, and recombination time is reduced by MXene through its influence on the recombination pathway. Since in both cases the samples containing 1 wt.% MXene achieved the highest photocatalytic efficiency, it can be concluded that a minimal amount of cocatalyst is most suitable. This conclusion is supported by the relatively larger TiO_2_–MXene contact area, and the other reason is the reduced TiO_2_ content in the samples. During the cycling experiments, a decrease in the photocatalytic activity of the PM samples was observed over three cycles. The rate of decrease was found to slow down, leading to stabilization of the sample. This decrease is attributed to changes in the quantity and quality of the surface active sites, and the slight instability of MXene itself. According to scientific publications, MXene is capable of being transformed into TiO_2_, and the rate of this transformation is dependent on the conditions; therefore, a TiO_2_–MXene composite cannot be used indefinitely as a photocatalyst. Although no photocatalyst operates indefinitely, this still represents an obvious limiting factor. In the case of certain CD samples, an increase in photocatalytic activity was observed during cyclization. This increase can be explained by the gradual transformation of MXene into TiO_2_ at the interface (anatase formation), resulting in an extension of the TiO_2_ lattice. Through this process, the contact is strengthened as the particles grow together (intergrowing), thereby facilitating electron transfer; this is considered one of the most important observations.

During the ethanol conversion process, it was observed that none of the samples achieved the photocatalytic efficiency of pure TiO_2_ (PM0). The measured photocatalytic activity converged with ethanol adsorption. Since the photocatalyst is not in suspension but is immobilized on a surface, the available contact area is reduced, and the process becomes adsorption-limited. The determined specific surface area values help in understanding the extent of the observed adsorption; a higher specific surface area was measured for the sample where adsorption was greater. The role of MXene as an electron trap is also clearly demonstrated in the ALD sample. We also performed cyclization during ethanol conversion, and a slight but continuous decrease in photocatalytic activity was observed.

Table 4 contains some literature comparisons regarding the TiO_2_–MXene composite.

## 4. Materials and Methods

### 4.1. Materials

Titanium aluminium carbide (Ti_3_AlC_2_, 99.9%, Sigma–Aldrich, St. Louis, MO, USA), hydrogen fluoride (HF, 40%, Molar Chemicals, Halásztelek, Hungary), titanium dioxide (p25 TiO_2_, >99, Evonik, Essen, Germany), titanium (IV) chloride (TiCl_4_, >98%, Sigma–Aldrich), acetic acid (CH_3_COOH, 97%, Sigma–Aldrich), hydrogen chloride (HCl, 37%, Sigma–Aldrich), ammonium hydroxide (NH_4_OH, 25%, Thermo Scientific Chemicals, Waltham, MA, USA), nitrogen (N_2_, 4.5 purity (99.995%), Messer Hungary Ltd., Budapest, Hungary), Milli-Q (MQ) water. All chemicals were used without further purification.

### 4.2. Synthesis

#### 4.2.1. Ti_3_C_2_ MXene

Ti_3_C_2_ MXene was produced using a solvothermal method [33]. Ti_3_C_2_ MXene was produced using a solvothermal method. Ti_3_AlC_2_ was added to 25 mL of a 40 wt.% HF solution at a concentration of 100 g·L^−1^., and the mixture was stirred for 45 min to initiate the etching process. The solution was then placed in a Teflon-lined autoclave, which was maintained at 60 °C for 24 h. After heat treatment, the resulting black powder was washed with MQ-water and absolute ethanol, facilitated by vacuum-assisted filtration. Finally, the purified Ti_3_C_2_ MXene was dried overnight using a lyophilizer to preserve its structure and prevent aggregation.

#### 4.2.2. TiO_2_–Ti_3_C_2_ MXene Composites

Four approaches were used during the synthesis of the composites. Via the first method, physical mixtures were prepared using simple grinding in an agate mortar: the as-synthesized MXene was mixed with P25 TiO_2_ catalyst. The concentrations of the MXene components were the following: 0, wt.%, 1 wt.%, 5 wt.% and 10 wt.%. The samples produced in this way are named PM0, PM1, PM5, and PM10, where “PM” stands for “physical mixtures”, and “X” stands for the concentration of MXene in wt.%.

In the second method TiO_2_ was grown on the surface of MXenes in the same concentration values. First, the production of TiO_2_ will be described in the next four sentences. TiO_2_ was synthesized via a sol–gel route [58]. A total of 4 mL of TiCl_4_ was slowly added to 8 mL, 37 wt.% HCl under magnetic stirring and cooling using an ice bath. The resulting mixture was added dropwise to 120 mL of Milli-Q water. Gelation was induced by the gradual addition of 2.08 mL glacial acetic acid. After homogenization, the pH was adjusted to pH 8 using 25% NH_3_ solution. The mixture was aged for two days, dried at 80 °C, and calcined at 500 °C, under nitrogen atmosphere.

The TiO_2_–Ti_3_C_2_ MXene composite synthesis was similar to the previous one (synthesis of TiO_2_); the only difference is that MXene was added in the reaction beaker, before the addition of TiCl_4_ to the HCl solution. The percentages of the added MXene were the following: 0 wt%, 1 wt%, 5 wt% and 10 wt%. The samples produced in this way were coded as CD0, CD1, CD5, and CD10, where “CD” stands for “chemical deposition”, and “X” stands for the concentration of MXene in wt.%.

The third method was the deposition of TiO_2_ using ALD on an MXene layer (MXene layer was created via spray coating; coverage was 0.5 mg·cm^−1^). The deposition was carried out using a Beneq TFS-200 atomic layer deposition (ALD) system. The support was a 10 cm × 10 cm glass sheet. For all depositions, a high-aspect-ratio chamber was employed with a residence time of 4 s and 8 s N_2_ purging between each step, both surfaces covered with 3000 layers of TiO_2_. One step corresponds to one layer. The TiO_2_ films were deposited at 523 K using TiCl_4_ (PURATREM Alfa Aesar, 99,0% Ti) and Milli-Q water as precursors. The samples produced in this way were named T_ALD and T+M_ALD for TiO_2_ coating on the glass and TiO_2_ coating on the glass with MXene coating.

### 4.3. Characterisation

A Rigaku Miniflex II diffractometer (Rigaku, Neu-Isenburg, Germany) was used to determine the crystalline composition of the samples in the range of 5–85 2θ° at 20 °C. During the measurements the following parameters were used: λ_CuKα_ = 0.15406 nm, 40 kV, and 30 mA.

The presence of TiO_2_ in the composites prepared through the ALD method was proofed with Raman spectroscopy. Raman spectra were recorded with a Bruker Senterra II Raman microscope (Bruker Optics, Inc., Billerica, MA, USA). The excitation wavelength of the laser was 532 nm, and the intensity of the laser beam was 1 mW, with an integration time of 10 s (5 repetitions), a resolution of 4 cm^−1^, and an interferometer resolution of 0.5 cm^−1^.

The separation of TiO_2_ using ALD was verified by the measuring of energy dispersive X-ray spectra (EDX) using a Hitachi S-4700 scanning electron microscope (Hitachi, Tokio Japan) with a Röntec EDX detector at 10 keV.

A Bruker Vertex 70 FTIR instrument was used to record Fourier transform infrared (FTIR) spectra for the investigation of functional groups. The spectra were recorded between the 400 and 4000 cm^−1^ range with a resolution of 4 cm^−1^.

The morphology of the samples was studied using scanning electron microscopy (Hitachi S-4700 microscope), using 10 kV of acceleration and a secondary electron detector.

The specific surface areas of the samples were determined by nitrogen adsorption at 77 K using a BELCAT-A device (Microtrac Retsch GmbH, Duesseldorf, Germany). The specific surface area was calculated via the BET method.

Diffuse reflectance spectra (DRS) were recorded between 200 and 800 nm using a Shimadzu UV-3600 Plus UV–VIS–NIR (Shimadzu Corporation, Kyoto, Japan) spectrophotometer with integration sphere for optical characterization. To obtain the band gap value the Kubelka–Munk method was used [59].

The room temperature steady-state photoluminescence (PL) spectra were measured using a Horiba Jobin Yvon Fluoromax-4 spectrofluorometer (Horiba, Kyoto, Japan) at 370 nm excitation wavelength, a band-pass filter was used to monochromatize the excitation light source, and a high-pass filter was used during emission measurement to exclude the excitation light.

The photocurrent density measurements were performed in a classic three-electrode cell using the linear sweep voltammetry (LSV) method at a sweeping rate of 2 mV·s^−1^ between −0.6 V and 0.2 V potential. The working electrode was an FTO plate spray-coated with the sample (1 mg·cm^−2^ coverage), the counter electrode was a platinum wire, and the reference electrode was an Ag/AgCl (in 3 mol·L^−1^ NaCl) electrode. The instrument used was a Metrohm Autolab PGSTAT302n potentiostat/galvanostat (Metrohm AG, Herisau, Switzerland). The experiments were carried out in a 0.5 mol·L^−1^ Na_2_SO_3_ solution, which ensured conductivity and oxidized as a hole acceptor on the working electrode, while H_2_ evolution occurred on the counter electrode. The intensity of the incoming light was 200 mW·cm^−2^. The spectrum of the light source used is shown in Figure S6.

### 4.4. Photocatalytic Activity Measurements

The photocatalytic activity of the produced composite photocatalysts and their pristine components was compared through the conversion of phenol. The experiments were conducted in a double-walled quartz cylinder surrounded by six fluorescent tubes, each with a power of 6 W, the spectrum of the light source is in Figure S7. The sample was homogenized beforehand using ultrasound, then kept in a homogeneous state with a magnetic stirrer and continuous air supply, which also helped to maintain a constant dissolved oxygen level. The temperature was maintained at 25 °C by circulating water in the reactor wall. Before starting the experiment, the suspension was kept in the dark for 15 min until the adsorption/desorption equilibrium was established. A photocatalytic experiment lasted 240 min, with a photocatalyst concentration of 1 g·L^−1^ in a volume of 100 mL and an initial phenol concentration of 10^−4^ mol·L^−1^. The phenol concentration of the collected samples was measured after centrifugation and filtration using a Merck-Hitachi L-7100 HPLC (Merck-Hitachi l-4250 UV–Vis detector and a Lichrospher Rp 18 column). The composition of the eluent used was 35/65 methanol/water, the flow rate was 0.8 mL·min^−1^, and the detection wavelength was 210 nm.

The photocatalytic efficiency of the samples and the electron-trapping properties of MXene were also measured at the solid/gas interface. To perform the tests, photocatalytic conversion of vapor-phase ethanol was carried out. Ethanol is an excellent model for VOCs (Volatile Organic Compounds) pollution, which is present in the air. The photocatalytic activity of TiO_2_/MXene layered composites produced using the ALD method was only examined using this method. The 10 cm × 10 cm surfaces produced were placed in a circulation reactor (the volume of the reactor was 165 cm^3^), which was closed by a 2 mm thick quartz window. The reactor was connected to a Shimadzu GC-14B (Shimadzu Corporation, Kyoto, Japan) gas chromatograph equipped with a thermal conductivity (TCD) and a flame ionization (FID) detector. The light source was placed 5 cm away from the sample surface, and the spectrum is shown in Figure S8. A total of 5 µL of ethanol and 2.5 µL of water were placed in the reactor, which completely vaporized at the beginning of the 30 min dark adsorption period. The initial concentration of the ethanol was 0.36 ± 0.018 mmol·L^−1^. A photocatalytic experiment lasted for 120 min after the light source was turned on. Samples taken periodically were fed directly into the gas chromatograph to measure the ethanol concentration. The column used was an Hayesep Q column, the eluent was 5.0 N_2_ gas, and the flow rate was 60 mL/min.

## 5. Conclusions

TiO_2_–MXene composites were synthesized using three different methods. The results indicate that the MXene concentration influences the structure of TiO_2_ obtained via the sol–gel method. Optical characterization (DRS and PL) revealed that the presence of MXene affects charge transport and recombination processes. Through the formation of a Schottky barrier, MXene can accept excited electrons from TiO_2_. Moreover, due to its surface functional groups, MXene affects the recombination of TiO_2_’s excited electrons by altering shallow defects. Both mechanisms enhance the utilization of TiO_2_ charge carriers during photocatalysis; in this study, the photon-generated hole predominantly governs the photocatalytic activity. The electron-trapping capability of MXene is further evidenced by measurements of photocurrent density. When applied in suspension, MXene enhanced the photocatalytic activity of TiO_2_. The results indicate that 1 wt.% represents the optimal MXene concentration in the composite; the total phenol content was converted in 40 min in the case of PM1. In contrast, when the photocatalyst was employed as a layer at the solid–gas interface, pure TiO_2_ exhibited the highest effectiveness, as its surface showed the greatest adsorption capacity. Overall, it can be concluded that the formation of TiO_2_–MXene composites enhances the photocatalytic activity of TiO_2_ under the investigated conditions.

## Figures and Tables

**Table 1:** Specific surface area of the samples.

Sample	Specific Surface Area (m^2^·g^−1^)
MXene	4.64
PM0	50.32
CD0	39.49
CD1	40.40
CD5	31.48
CD10	24.60

**Table 2:** The normalized phenol and ethanol conversion after 40 (phenol) and 10 (ethanol) minutes.

Sample	Converted Phenol (mol·g^−1^·min^−1^)			Converted Ethanol (mol·g^−1^·min^−1^)		
	Cycle 1	Cycle 2	Cycle 3	Cycle 1	Cycle 2	Cycle 3
PM0	2.07 × 10^−6^	1.95 × 10^−6^	1.93 × 10^−6^	2.98 × 10^−5^	2.93 × 10^−5^	2.83 × 10^−5^
PM1	2.46 × 10^−6^	1.46 × 10^−6^	1.51 × 10^−6^	1.13 × 10^−5^	8.12 × 10^−6^	7.57 × 10^−6^
PM5	2.33 × 10^−6^	2.21 × 10^−6^	2.14 × 10^−6^	8.50 × 10^−6^	6.70 × 10^−6^	6.49 × 10^−6^
PM10	2.22 × 10^−6^	2.17 × 10^−6^	2.09 × 10^−6^	7.26 × 10^−6^	5.21 × 10^−6^	6.49 × 10^−6^
CD0	2.85 × 10^−7^	1.95 × 10^−7^	1.91 × 10^−7^	1.05 × 10^−5^	9.46 × 10^−6^	7.95 × 10^−6^
CD1	7.17 × 10^−7^	7.11 × 10^−7^	7.18 × 10^−7^	1.03 × 10^−5^	6.47 × 10^−6^	6.23 × 10^−6^
CD5	3.06 × 10^−7^	3.95 × 10^−7^	5.14 × 10^−7^	7.86 × 10^−6^	6.22 × 10^−6^	5.51 × 10^−6^
CD10	3.01 × 10^−7^	3.57 × 10^−7^	3.85 × 10^−7^	8.84 × 10^−6^	6.68 × 10^−6^	5.66 × 10^−6^
T_ALD	-	-	-	8.20 × 10^−7^	4.60 × 10^−7^	2.61 × 10^−7^
T+M_ALD	-	-	-	2.21 × 10^−6^	1.54 × 10^−6^	1.39 × 10^−6^

**Table 3:** The relative adsorption/absorption rate after 30 min.

Sample	Relative Adsorbed/Absorbed Ethanol (%)
MXene	3.18
PM0	33.41
PM1	9.07
PM5	24.92
PM10	8.66
CD0	22.11
CD1	13.53
CD5	21.19
CD10	11.99
T_ALD	1.71
T+M_ALD	0.83

**Table 4:** Comparison of photocatalytic efficiency with published data in the literature.

Photocatalyst	Pollutant	Efficiency	References
TiO_2_/Ti_3_C_2_T_x_	methylorange	92% (50 min)	[51]
MoS_2_@Ti_3_C_2_	methylorange	98% (60 min)	[52]
AgNPs/TiO_2_/Ti_3_C2T_x_	methylene blue	99% (30 min)	[53]
Ti_3_C_2_/g-C_3_N_4_	methylene blue	100% (180 min)	[54]
TiO_2_@Ti_3_C_2_	rhodamine B	97% (40 min)	[55]
BiVO_4_/Ti_3_C_2_	Congo red	99.5% (60 min)	[56]
TiO_2_/Ti_3_C_2_/MnFeO_4_	carbamazepine	100% (60 min)	[57]
TiO_2_/Ti_3_C_2_/MnFeO_4_	ibuprofen	100% (60 min)	[57]
TiO_2_/Ti_3_C_2_	ethanol	84% (90 min)	this study
TiO_2_/Ti_3_C_2_	phenol	99% (40 min)	this study

## Data Availability

The original contributions presented in this study are included in the article. Further inquiries can be directed to the corresponding authors.

## Supplementary Materials

The following supporting information can be downloaded at https://www.mdpi.com/article/10.3390/ijms27093975/s1.
