# Supplementary material for: MXene/TiO2 Photocatalyst: The Key Role of MXene Electron Trapping in Water and Air Treatment
Source: Int J Mol Sci. 2026 Apr 29;27(9):3975. doi: 10.3390/ijms27093975 (PMC13164373; doi:10.3390/ijms27093975)
Supplement: Supplementary file 1 [file ijms-27-03975-s001.zip › ijms-4227736-supplementary.pdf]

Supporting Information

MXene/TiO_2 Photocatalyst: The Key Role of MXene Electron Trapping in Water and Air Treatment

Áron Ágoston [1,2,*], Laura Lakatos [3], Ágota Deák [1,*], Gergő Ballai [3], Karolina Solymos [4], Szabolcs Kocsis Szürke [5], László Janovák [1], Ákos Kukovecz [3], Zoltán Kónya [3], Zsolt Pap [3,6,7]

1 Department of Physical Chemistry and Materials Sciences, University of Szeged, Aradi v.sqr.1, H-6720 Szeged, Hungary; janovakl@chem.u-szeged.hu

2 Interdisciplinary Excellence Center, Department of Physical Chemistry and Materials Science, University of Szeged, Rerrich B. sq. 1, H-6720 Szeged, Hungary

3 Department of Applied and Environmental Chemistry, University of Szeged, Rerrich Béla sqr. 1, H-6720 Szeged, Hungary; laura.lakatos@chem.u-szeged.hu (L.L.); ballaig@chem.u-szeged.hu (G.B.); kakos@chem.u-szeged.hu (Á.K.); konya@chem.u-szeged.hu (Z.K.); pzsolt@chem.u-szeged.hu (Z.P.)

4 Department of Physical and Environmental Geography, University of Szeged, Egyetem Str. 2-6, H-6722 Szeged, Hungary; solymoskarolina@geo.u-szeged.hu

5 Central Campus Győr, Széchenyi István University, Egyetem sqr 1. 303., H-9026 Győr, Hungary; kocsis.szabolcs@ga.sze.hu

6 Centre 3B, Laboratory of Advance Hydrobiology and Biomonitoring, Babeş-Bolyai University, Clinicilor 5–7, R-400015 Cluj-Napoca, Romania

7 Hungarian Department of Biology and Ecology, Faculty of Biology and Geology, Babeş-Bolyai University, Republicii 44, R-400015 Cluj-Napoca, Romania

* Correspondence: agostona@chem.u-szeged.hu (Á.Á.); agotadeak@chem.u-szeged.hu (Á.D.); Tel.: +36-62-543-012 (Á.Á.); +36-62-544-211 (Á.D.)

Glass support

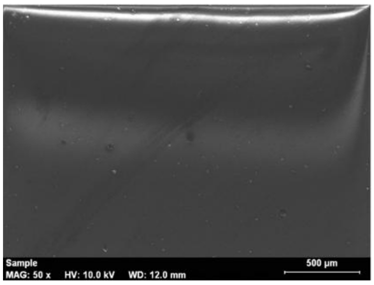

Element	norm. wt. %	at. %	Error in [%]
Carbon	0.000	0.000	0.001
Oxygen	62.270	73.615	12.965
Sodium	9.019	7.420	1.676
Magnesium	2.284	1.778	0.671
Aluminium	0.565	0.396	0.559
Silicon	22.755	15.325	3.736
Calcium	3.107	1.466	1.736

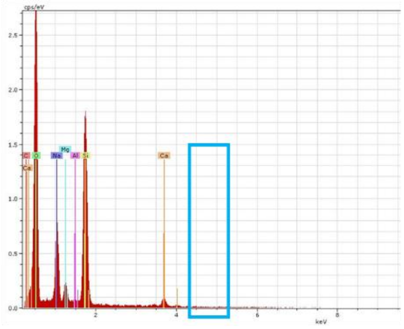

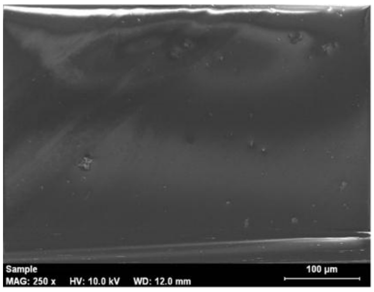

Element	norm. wt. %	at. %	Error in [%]
Carbon	0.000	0.000	0.001
Oxygen	72.626	81.449	14.781
Sodium	8.437	6.585	1.683
Magnesium	1.805	1.332	0.746
Aluminium	0.599	0.398	0.657
Silicon	14.822	9.470	2.761
Calcium	1.712	0.766	2.100

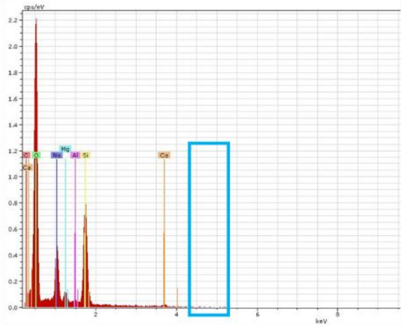

Glass support + TiO2 (3000 layer ALD)

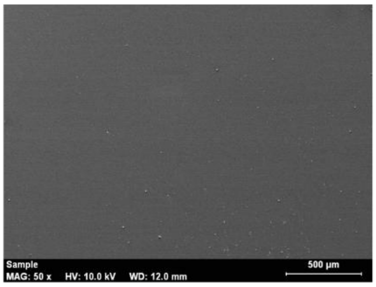

Element	norm. wt. %	at. %	Error in [%]
Carbon	0.490	1.087	0.418
Oxygen	38.801	64.626	7.523
Sodium	0.314	0.364	0.557
Magnesium	0.151	0.165	0.329
Aluminium	0.173	0.171	0.347
Silicon	0.320	0.304	0.519
Calcium	0.253	0.168	0.470
Titanium	59.498	33.115	12.325

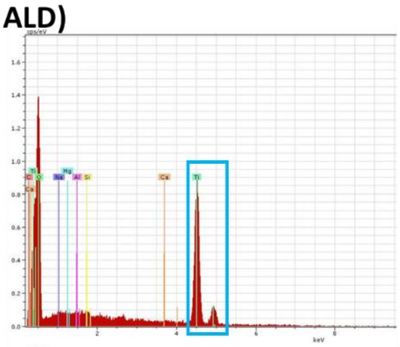

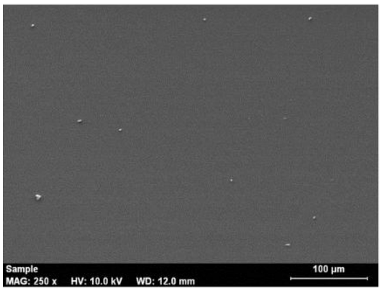

Element	norm. wt. %	at. %	Error in [%]
Carbon	0.476	1.057	0.411
Oxygen	38.819	64.643	7.583
Sodium	0.403	0.467	0.665
Magnesium	0.154	0.169	0.333
Aluminium	0.128	0.126	0.292
Silicon	0.296	0.281	0.491
Calcium	0.226	0.150	0.438
Titanium	59.498	33.108	12.428

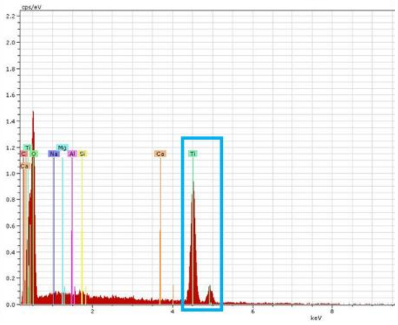

Figure S1. The image of the measured surface and the ED spectrum of the elemental composition determined during the measurement).

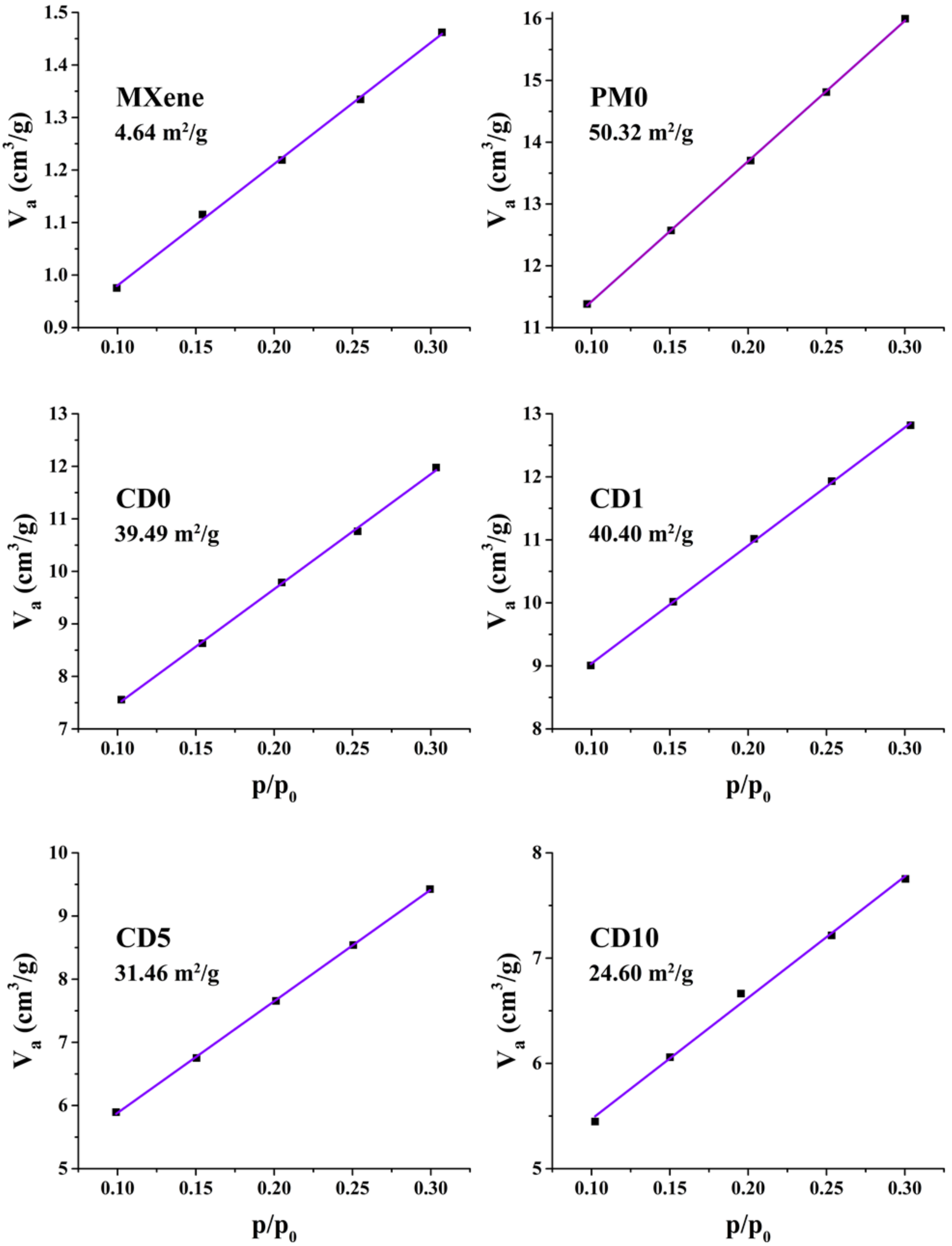

Figure S2. The BET plot of the samples.

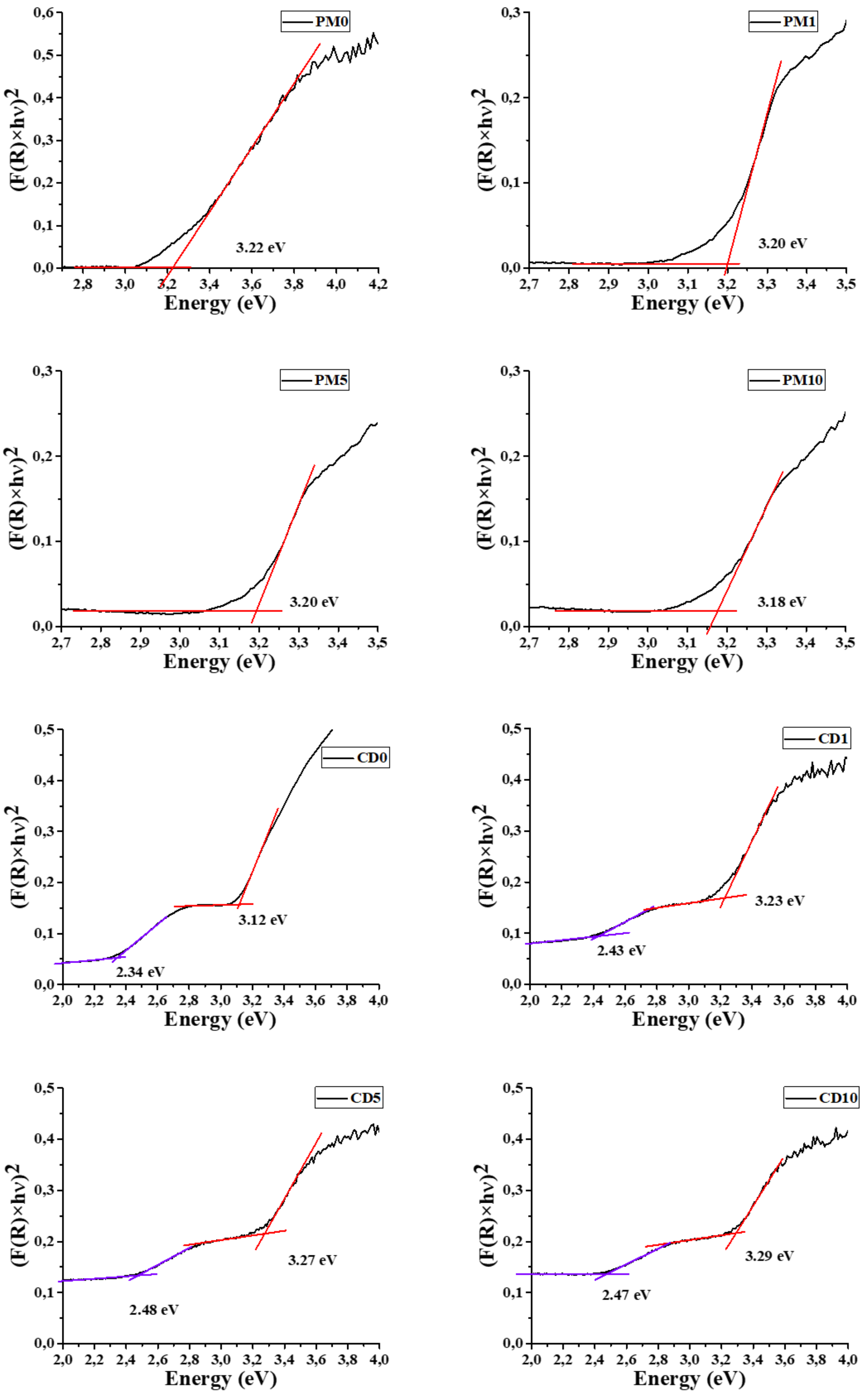

Figure S3. Determination of band gap of the prepared samples.

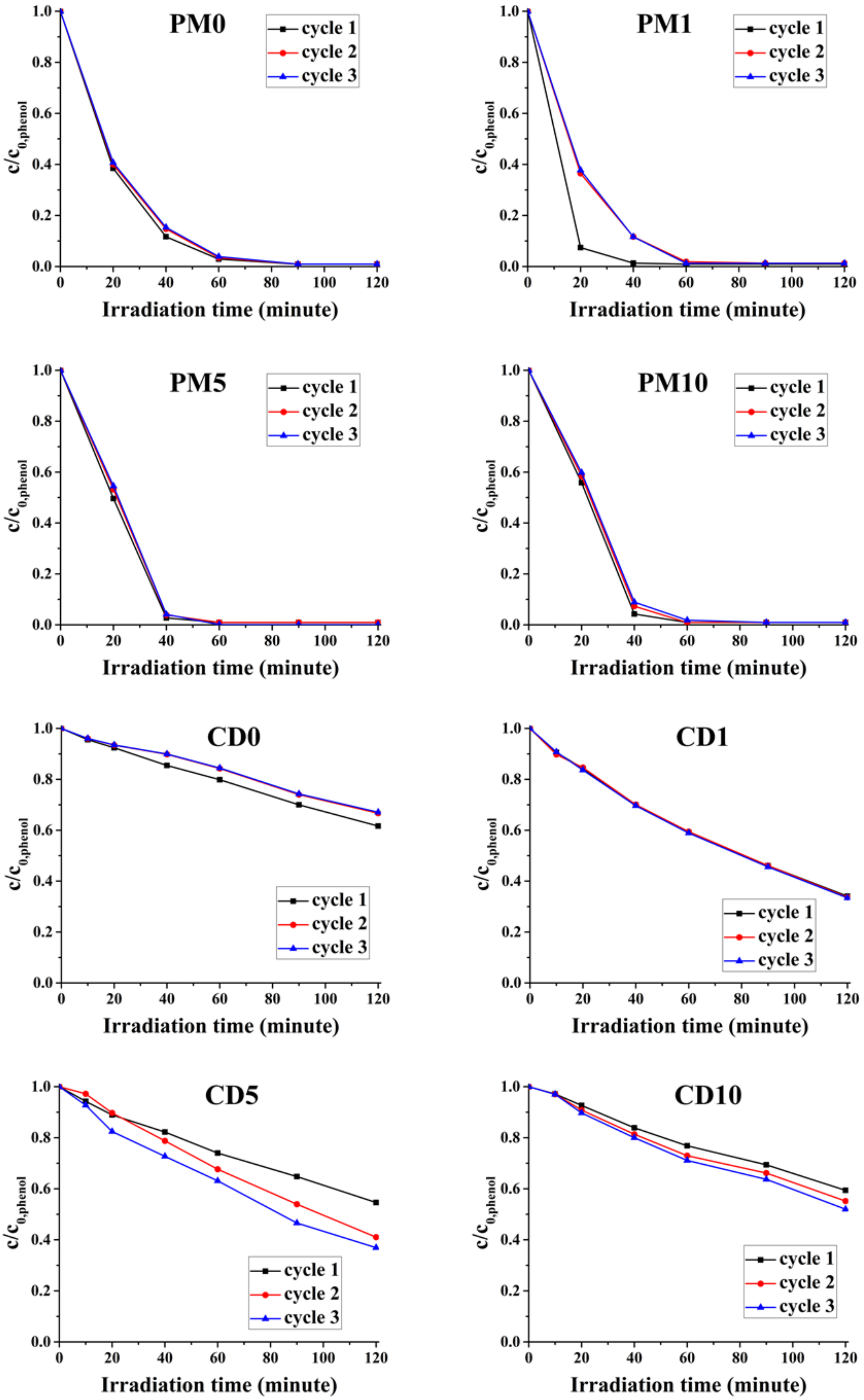

Figure S4. Photocatalyst stability, the cyclization of photocatalytic activity (phenol).

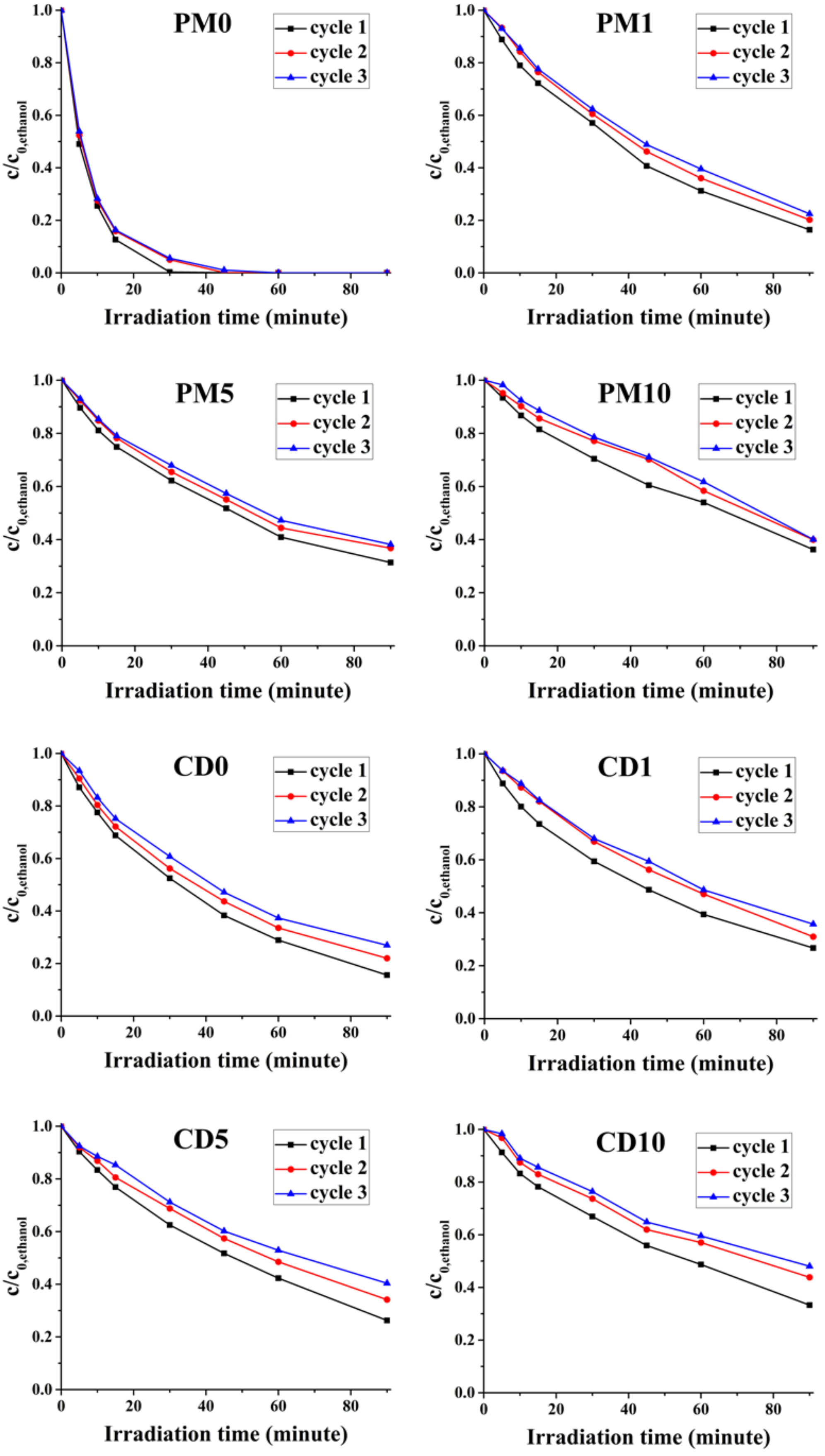

Figure S5. Photocatalyst stability, the cyclization of photocatalytic activity (ethanol).

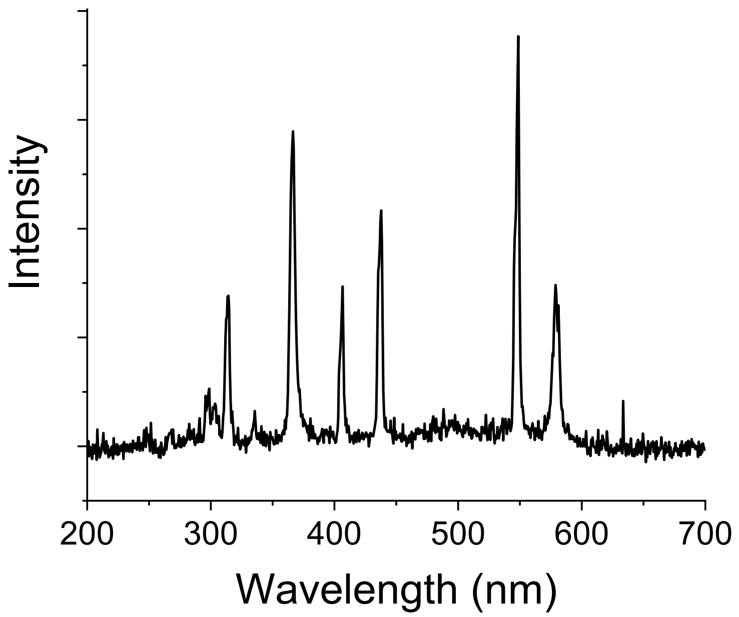

Figure S6. Emission spectrum of the light source used during the photocurrent measurements.

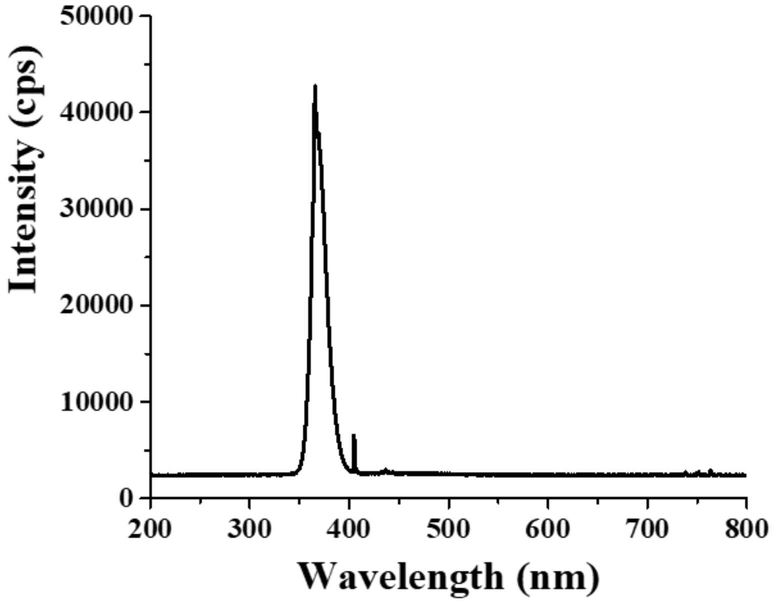

Figure S7. Emission spectrum of the light source used during photocatalytic oxidation experiments in suspension (S/L interface) (phenol conversion).

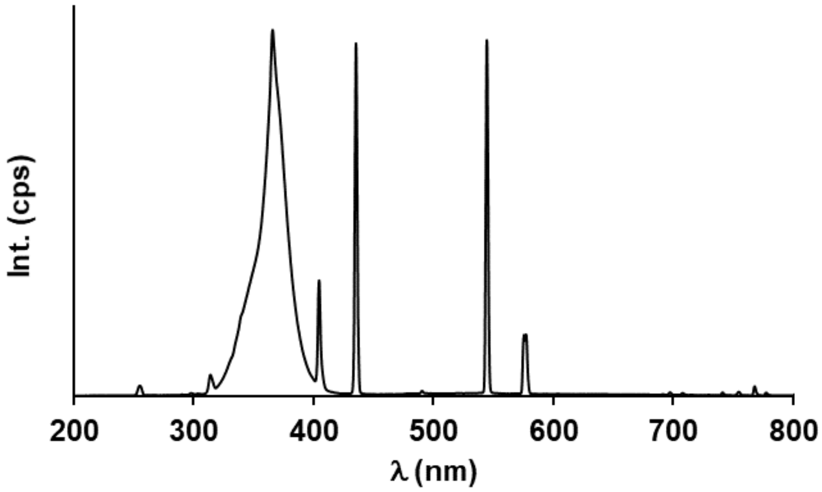

Figure S8. Emission spectrum of the light source used during photocatalytic oxidation experiments on S/G interface (ethanol conversion).
